# Carbon-Coated Magnetic Nanoparticle Dedicated to MRI/Photoacoustic Imaging of Tumor in Living Mice

**DOI:** 10.3389/fbioe.2021.800744

**Published:** 2021-12-02

**Authors:** Yujing Li, Fei Ye, Shanxiang Zhang, Wenjun Ni, Liewei Wen, Huan Qin

**Affiliations:** ^1^ MOE Key Laboratory of Laser Life Science and Institute of Laser Life Science, Guangdong Provincial Key Laboratory of Laser Life Science, College of Biophotonics, College of Biophotonics, South China Normal University, Guangzhou, China; ^2^ Zhuhai Precision Medical Center, Zhuhai People’s Hospital, Zhuhai Hospital Affiliated with Jinan University, Zhuhai, China; ^3^ Guangzhou Key Lab of Spectral Analysis and Functional Probes, College of Biophotonics, South China Normal University, Guangzhou, China

**Keywords:** carbon-coated magnetic nanoparticle, multimodality imaging, photoacoustic imaging, magnetic resonance imaging, glioblastoma

## Abstract

Multimodality imaging can reveal complementary anatomic and functional information as they exploit different contrast mechanisms, which has broad clinical applications and promises to improve the accuracy of tumor diagnosis. Accordingly, to attain the particular goal, it is critical to exploit multimodal contrast agents. In the present work, we develop novel cobalt core/carbon shell–based nanoparticles (Cobalt at carbon NPs) with both magnetization and light absorption properties for dual-modality magnetic resonance imaging (MRI) and photoacoustic imaging (PAI). The nanoparticle consists of ferromagnetic cobalt particles coated with carbon for biocompatibility and optical absorption. In addition, the prepared Cobalt at carbon NPs are characterized by transmission electron microscope (TEM), visible–near-infrared spectra, Raman spectrum, and X-ray powder diffraction for structural analysis. Experiments verify that Cobalt at carbon NPs have been successfully constructed and the designed Cobalt at carbon NPs can be detected by both MRI and PAI *in vitro* and *in vivo*. Importantly, intravenous injection of Cobalt at carbon NPs into glioblastoma-bearing mice led to accumulation and retention of Cobalt at carbon NPs in the tumors. Using such a multifunctional probe, MRI can screen rapidly to identify potential lesion locations, whereas PAI can provide high-resolution morphological structure and quantitative information of the tumor. The Cobalt at carbon NPs are likely to become a promising candidate for dual-modality MRI/PAI of the tumor.

## Introduction

Complete surgical resection is the primary method for most solid tumors. However, because of the aggressive growth of tumor cells, clinicians face significant challenges in identifying and completely removing cancer tissue that is scattered sporadically (Baroncini et al., 2007; Issard et al., 2021). Furthermore, residual tumor tissues after surgery probably that are triggered usually leads to cancer recurrence and adverse sequelae. For achieving more complete tumor resections, many efforts have been made to explore many techniques in tumor imaging. For instance, magnetic resonance imaging (MRI) has been used to guide stereotactic surgical removal of tumors for preoperative planning (He et al., 2017; Ding et al., 2019; Wang et al., 2019; Liu et al., 2019; Bucci et al., 2004). However, because of tissue displacement, the tumor boundary delineated by this method in preoperative MRI is inconsistent with the actual tumor boundary during surgery (Reinges et al., 2004); consequently, the guidance of surgical resection by preoperative localization alone is limited.

Intraoperative imaging exhibits favorable potential in guiding tumor resection by improving the ratio of target background to distinguish tumor tissues (Chi et al., 2014). In the intraoperative imaging modalities used for image-guided resection, optical imaging based on tissue intrinsic optical properties or exogenous contrast agents has the advantages of fast acquisition speed, high sensitivity, convenient operation, and affordable running cost. However, because of strong light scattering, the resolution and penetration depth of these optical imaging techniques are limited, ultimately limiting the detection and localization of tumors below the surface (Razansky et al., 2009; Niu et al., 2012; Chen et al., 2017).

Photoacoustic imaging (PAI) overcomes the limitation of light diffusion by combining photoexcitation with photoacoustic detection to realize deeper target imaging with high spatial resolution (Xu and Wang, 2006; Wang and Hu, 2012; Hu et al., 2019; Kim et al., 2010). It uses laser pulses to irradiate a target sample, which absorbs the laser energy, resulting in rapid thermoelastic expansion of the absorber that excites a wide-band ultrasound wave. A back-projected reconstruction algorithm is used to reconstruct a map of the distribution of the optical energy deposition within the living in living subjects (Yin et al., 2004; Yang et al., 2005; Yang et al., 2007; Yang et al., 2007; Yang et al., 2008; Xiang et al., 2009). PAI is a non-invasive imaging method that depends on the differences in the light absorption coefficients of biological tissues. In biological tissues, PA signals vary with the distribution of light absorption. Thus, PAI resembles to optical imaging in that it has a high contrast and sensitivity to tissue features. (Huang et al., 2013; Qin et al., 2013; Zhang et al., 2014; Qin et al., 2015). However, in PAI technology, the carrier of light absorption characteristics is not light signal but ultrasonic signal, and the penetration of sound to biological tissues is several orders of magnitude higher than that of light. As a result, PAI offers a far greater depth of penetration and scalable spatial resolution than other optical imaging methods (Razansky et al., 2009; Wang and Yao, 2016; Wang, 2009; Liang et al., 2021; Cheng et al., 2020).

Distinguishing tumors, especially tumors at the early stage, is extremely difficult using an imaging system only. Accurate localization and resection of tumor by imaging probe is very important for improving surgical prognosis. In particular, with the help of multimodal imaging probes, it is able to provide more comprehensive image of tumors benefiting from the contrast improvement (Lv et al., 2020). The development of a dual-channel MRI and PAI probe can be isolated by the tumor and retained long enough, so that a single injection of this drug will aid in intraoperative resection and preoperative planning of the tumor, even subtissue tumors. (Liao et al., 2019). Recently, cobalt-based nanoparticles have being extensively investigated for MRI and PAI contrast agents, owing to great optical absorption properity, magnetocrystalline anisotropy, and large magnetostrictive coefficient. Nevertheless, it is still a bottleneck problem to improve the biocompatibility of cobalt-based nanoprobes and the efficacy of dual-modality imaging to be more conducive to their clinical transformation.

**FIGURE 1:**
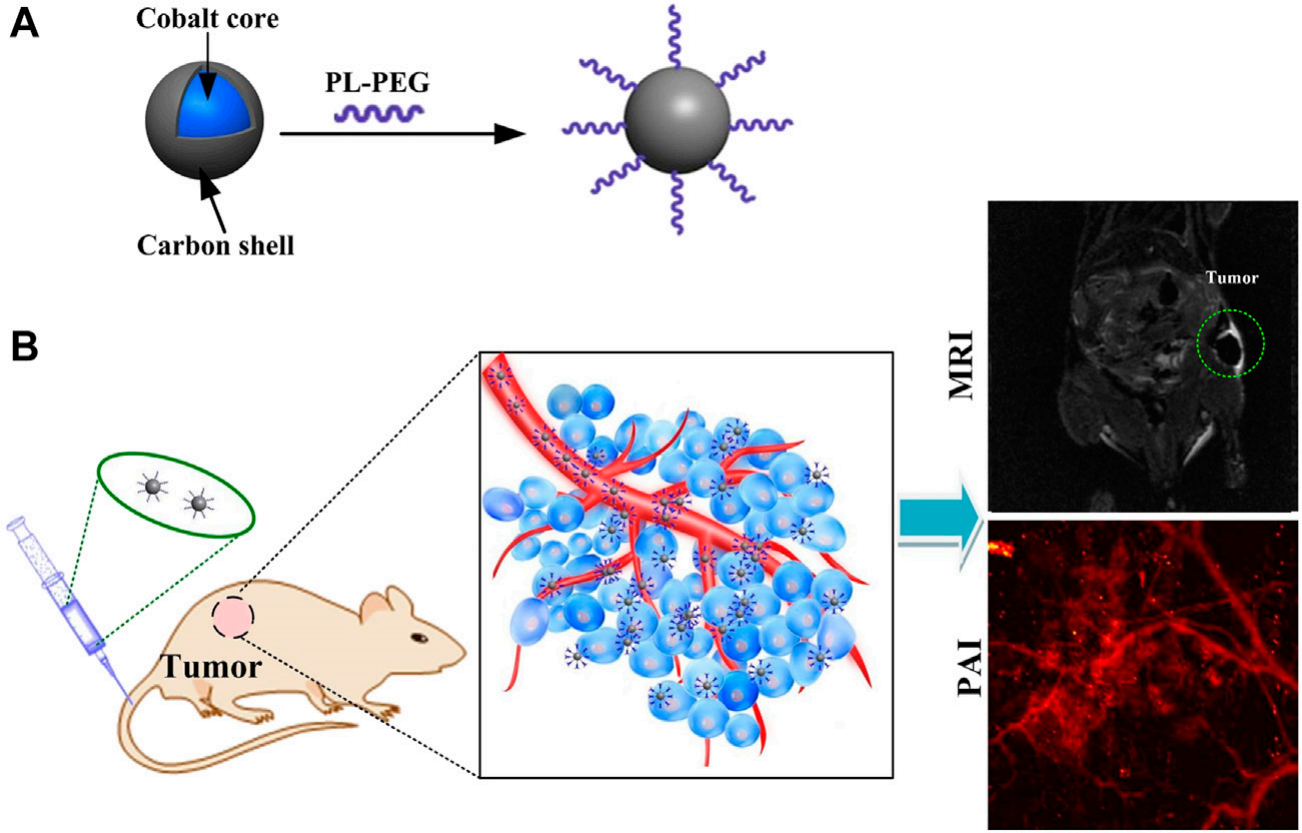
**(A)** Schematic diagram of the surface modification and preparation of the cobalt core/carbon shell nanoparticles (Cobalt at carbon NPs). **(B)** Schematic illustration of tumor-targeted dual-modality MR/photoacoustic imaging of Cobalt at carbon NPs.

Here, we have developed novel cobalt core/carbon shell–based nanoparticles (Cobalt at carbon NPs) for dual-modality MRI and PAI. To improve biocompatibility and absorption of light, nanomaterials are composed of cobalt particles coated with carbon (Figure 1A). The designed Cobalt at carbon NPs can be detected by both MRI and PAI *in vitro* or *in vivo*. After intravenous injection of cobalt at carbon nanotubes into mice carrying glioblastoma, the enhanced permeation and retention (EPR) effect caused tumor accumulation and retention of Cobalt at carbon nanotubes, allowing the use of two-channel non-invasive tumor detection. With Cobalt at carbon NPs, there is great potential to develop multiscale complementary imaging schemes. MRI can identify the location of tumor for preoperative planning, and high spatial resolution PAI provides subsequent precise blood vessel morphology and tumor imaging from the surface to the depths to accurately guiding tumor resection (Figure 1B).

## Results

### Synthesis and Characterization of Cobalt at Carbon NPs

**FIGURE 2:**
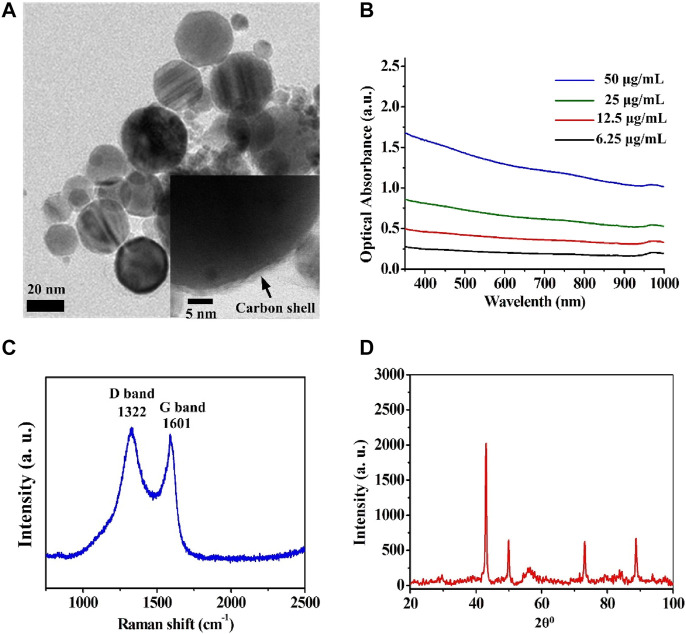
Structural analysis of the Cobalt at carbon NPs. **(A)** Transmission electron microscope (TEM) images of the Cobalt at carbon NPs. **(B)** UV/visible–near-infrared spectra of the Cobalt at carbon NPs with different concentrations in solution (6.25, 12.5, 25, and 50 μg/ml). **(C)** Raman spectrum (excitation 1,064 nm) of the Cobalt at carbon NPs, showing the G and D bands of carbon. **(D)** Powder XRD data for nanocrystals.

Cobalt nanoparticles cannot be used as MRI contrast agents on account of their oxidation-induced instability and toxicity. However, carbon coating has good biocompatibility and good air isolation, which can overcome the above shortcomings of cobalt nanoparticles. Furthermore, carbon enables optical absorption that can be used in PAI. To analyze the structure of Cobalt at carbon NPs, a series of verification experiments were performed. Transmission electron microscope (TEM) revealed that the average size of the Cobalt at carbon NPs was less than 50 nm (Figure 2A). The thickness of the carbon shell is about 3 nm (insert in Figure 2A). UV/visible–near-infrared spectra of the Cobalt at carbon NPs with different concentrations in solution (6.25, 12.5, 25, and 50 μg/ml) shown that Cobalt at carbon NPs exhibit optical response (Figure 2B), making it amenable to PAI studies. A Raman spectrum (excitation 1,064 nm) of the Cobalt at carbon NPs show the G and D bands of carbon (Figure 2C), providing evidence for the graphitic shell (Tuinstra and Koenig, 1970). We identified a crystalline body-centered-cubic Cobalt at carbon core for nanocrystals by powder X-ray diffraction (XRD, Figure 2D). As shown in powder-XRD data for nanocrystals (Figure 2D). The small peaks marked with an asterisk on the XRD curve of the nanocrystals correspond to face-centered-cubic Cobalt. These experiments demonstrated that Cobalt at carbon NPs have been successfully constructed.

### PAI and MRI of Cobalt at Carbon NPs *in vitro*

**FIGURE 3:**
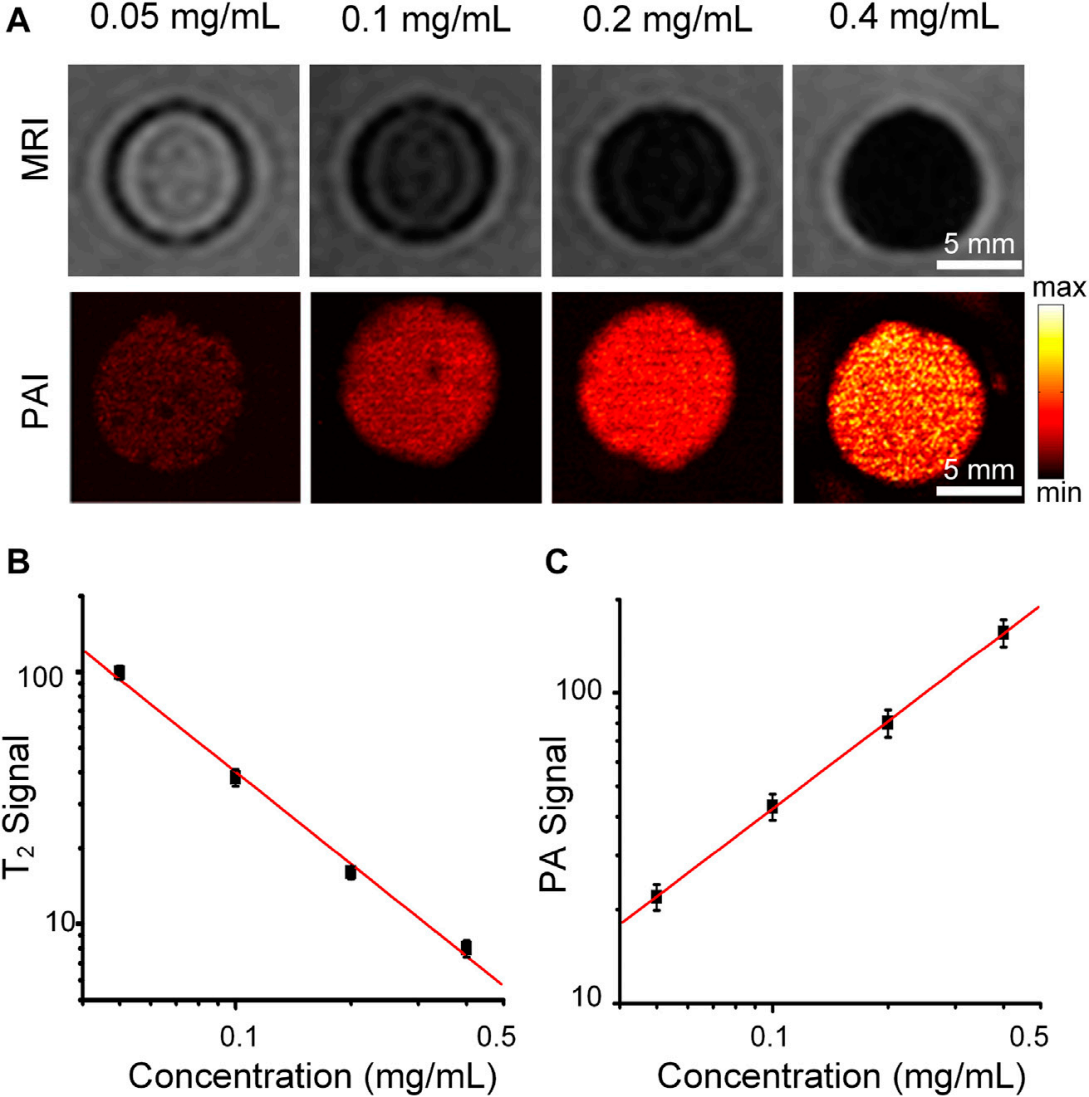
MRI and PAI of the Cobalt at carbon NPs phantom gels. **(A)** T_2_-weighted spin echo image and PA image of Cobalt at carbon NPs agarose gel phantoms. The concentrations of Cobalt at carbon NPs were 0.05, 0.1, 0.2, and 0.4 mg/ml, respectively. **(B)** T_2_ signal intensity of MRI and **(C)** PA signal intensity at different concentrations (0.05–0.4 mg/ml).

We further validated that Cobalt at carbon NPs can enhance both PAI and MRI contrast. T_2_-weighted spin echo image of Cobalt at carbon NPs agarose gel phantoms are shown in Figure 3A. T_2_-weighted spin echo images exhibit that higher concentrations bring about lower MRI signals, indicating shorter T_2_ values for water protons (Figure 3A). As the concentration of Cobalt at carbon NPs increased, MR signal intensity decreased (Figure 3B). The significant change in the MRI signal intensity can be attributed to ferromagnetic cobalt in the core of the nanoparticle. For the PAI test, some absorption-free and scatter-free agarose membranes containing cobalt at carbon nanotubes with concentrations increased from 0.05, 0.1, 0.2, and 0.4 mg/ml were constructed (n = 3 inclusions of each concentration). The PA signals generated by Cobalt at Carbon NPs were highly correlated with the concentration of nanoparticles (*R* ^2^ = 0.980, Figure 3C). In addition, PA imaging produced by gel blocks containing Cobalt at Carbon NPs has a high level of edge differentiation, indicating its potential to identify samples or tumor edges. These experiments demonstrated that the Cobalt at carbon NPs could be used for improving both MR and photoacoustic signals and have good MRI and PAI dual-mode imaging ability.

**The cellular uptake, and cells PAI of Cobalt at carbon NPs**

**FIGURE 4:**
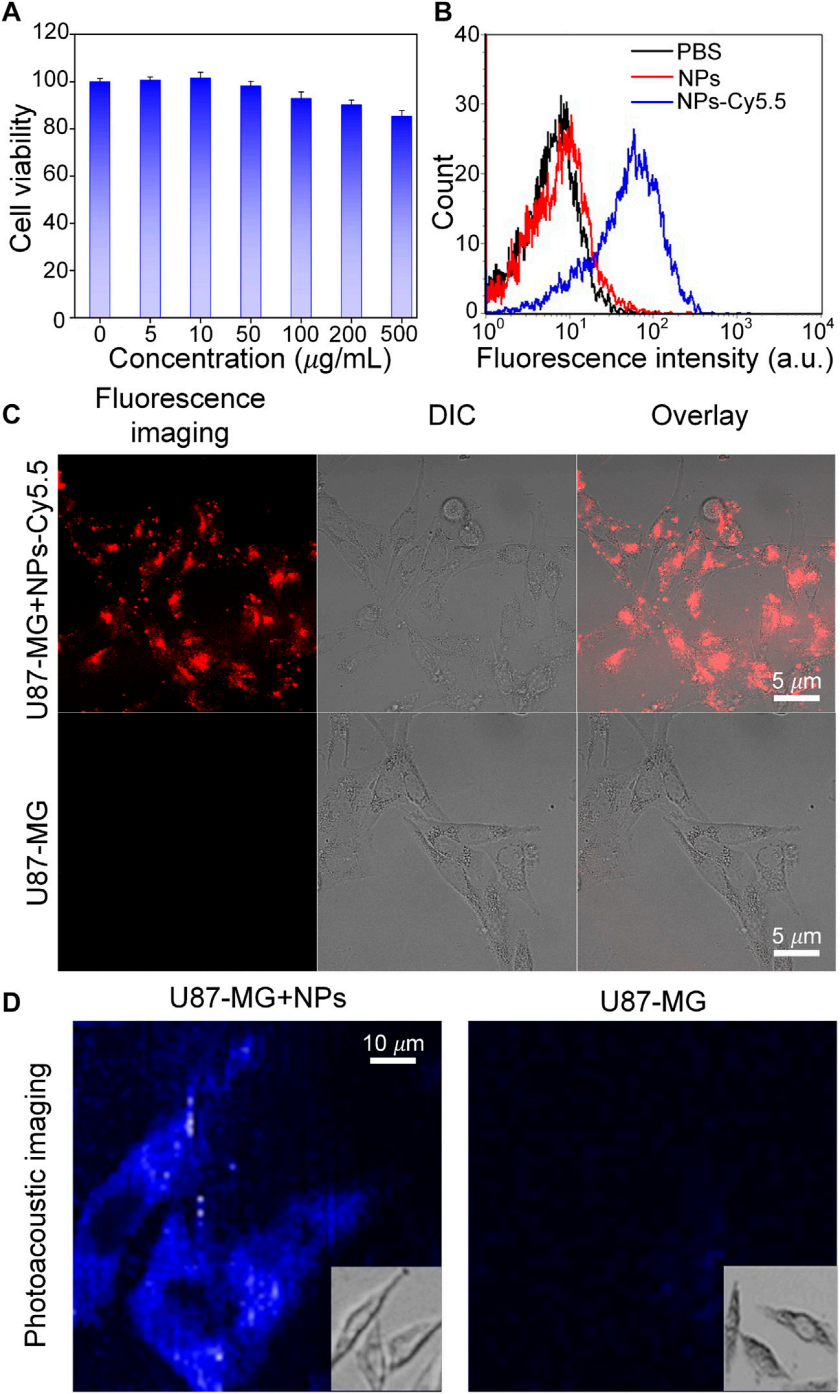
Experiments to test the targeting ability of Cobalt at carbon NPs *in vitro*. **(A)** After culture with various concentrations of Cobalt at carbon NPs for 24 h. **(B)** Flow cytometry analysis of U87-MG cells incubated with PBS, Cobalt at carbon NPs, and Cobalt at carbon NPs–Cy5.5. **(C)** Cell uptake of Cobalt at carbon NPs–Cy5.5 in U87-MG cells. The fluorescence images were taken by confocal microscopy. **(D)** *In vitro* PAI of U87-MG cells after 4 h treatment with Cobalt at carbon NPs.

To test whether Cobalt at carbon NPs can be taken up by U87 cells, first, the cell cytotoxicity of Cobalt at carbon NPs was measured using a standard CCK-8 assay. U87-MG glioma cells were used as experimental cell model. Primally, U87 glioma cells were co-incubated in cell culture medium with different concentrations (0, 5, 50, 100, 200, and 500 μg/ml) of Cobalt at carbon nanotubes for 24 h. The Cobalt at carbon NPs had no obvious toxic effect that affected the cytoactive of the U87-MG glioma cells (Figure 4A). Then, the subcellular localization of Cobalt at carbon NPs–Cy5.5 was investigated in cultured tumor cells. Nanoparticles were used to incubate U87-MG cells, whereas PBS was used to incubate U87 cells as a control, and the same treatment was then applied to all groups. Under confocal microscopy, fluorescence signals were detected on the cell membranes of Cobalt at carbon NPs–Cy5.5–incubated U87 cells (Figure 4C), whereas very small signals were detected in U87-MG cells incubated with PBS. Flow cytometry was used to further study the cell uptake characteristics. The cell uptake of Cobalt at carbon NPs–Cy5.5 was higher than that of PBS group (Figure 4B). These above results suggested that Cobalt at carbon NPs could be taken up by U87-MG cells.

To further prove that Cobalt at Carbon NPs can be taken up by U87-MG cells and have PAI ability on U87-MG cells, U87-MG cells were cultured with Cobalt at Carbon NPs (100 μg/ml) for 4 h, and PAI was performed as the control group. PAI was also performed on U87-MG cells cultured without Cobalt at carbon NPs. Figure 4D shows that U87-MG cells cultured with Cobalt at carbon NPs can be fully displayed by PAI, whereas cells in the control group cannot be observed with PAI. This further indicates that Cobalt at carbon NPs can be absorbed by U87-MG cells and have excellent PIA ability.

### MRI and PAI of Tumor *in vivo*

**FIGURE 5:**
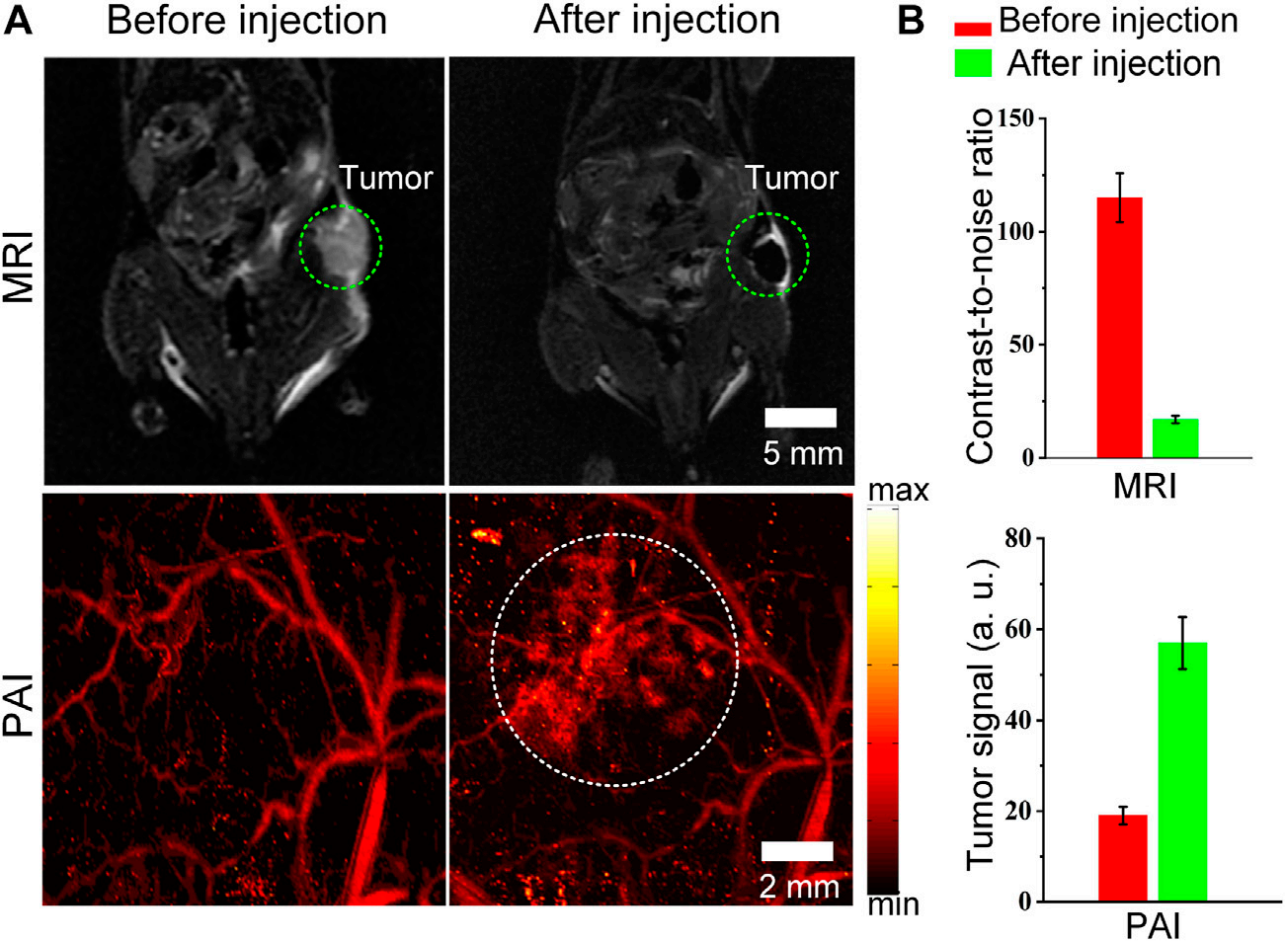
MRI and PAI detection of tumors in living mice with Cobalt at carbon NPs **(A)** Two-dimensional axial MRI and photoacoustic images. The post-injection images of both modalities showed clear tumor visualization **(B)** Quantification of the signals in the tumor showing a significant change in the MRI and PA signals after as compared to before the injection (n = 3 mice).

Finally, the advantage of combined MRI and PAI for tumor imaging *in vivo* is demonstrated. We attempted to test whether the Cobalt at carbon NPs could be used for tumor detection in living mice. Mice bearing U87 tumors (n = 3 mice) were used for both MRI. After validating that Cobalt at carbon NPs had no apparent toxicity at low concentration (1 mg/ml) *in vitro* (data not shown). The 200 μl of Cobalt at carbon NPs (2 mg/ml) were injected into mice by tail vein. Both MR and photoacoustic images of tumors before and after injection of Cobalt at carbon NPs were acquired. The low signal regions in the T_2_-weighted MR images indicate the locations of accumulated Cobalt at carbon NPs (Figure 5A). The nanoparticles accumulate within the tumor without necessitating a targeting mechanism that could be contributed to EPR effect, as new tumor vessels that sprout from existing vessels are often leaky, with large pores. Therefore, a high-resolution photoacoustic image of tumor was obtained. In the PA image of the tumor, the deformity of the vascular morphology was clearly shown (Figure 5A). At 6 h after injection of Cobalt at carbon NPs, the PA signal around the blood vessels increased, which indicated that the Cobalt at carbon NPs permeating from the blood vessels and are retention in the tumors (Figure 5A). The quantification of tumor signals in areas of interest showed significant changes in both MRI and PAI compared to pre-caudal vein injection (Figure 5B). The MRI contrast-to-noise ratio decreased from 115 ± 10.7 to 18 ± 1.65 AU. The PA signal improved by ∼96%, from 29 ± 2.95 to 57 ± 5.8 AU. The results demonstrate that Cobalt at carbon NPs could accumulate and detain in the tumors, allowing for multiscale tumor imaging using MRI and PAI. With Cobalt at carbon NPs, a multi-scale complementary imaging protocol can be established: MRI can discern the location of tumor for preoperative planning, and high spatial resolution PAI provides subsequent precise blood vessel morphology and tumor imaging from the surface to the depths to accurately guide tumor resection.

### *In vivo* Photothermal Therapy Potential

**FIGURE 6:**
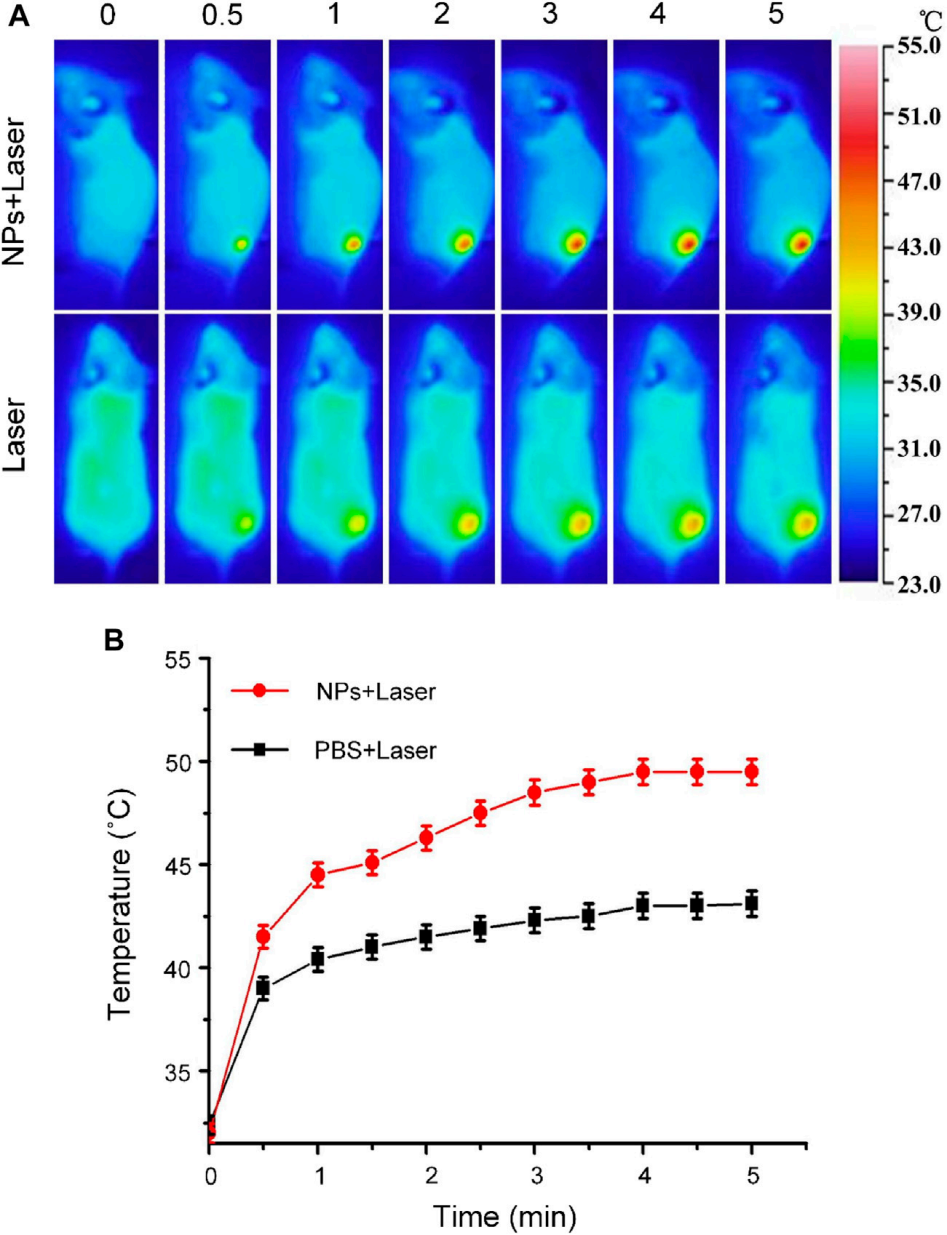
**(A)** Infrared thermographic maps and **(B)** Time-dependent temperature increase in the U87-MG glioma tumor-bearing nude mice irradiated by the 808-nm laser (1 W/cm^2^) at 6 h after separate intravenous injection with 100 μl of PBS and Cobalt at carbon NPs (1 mg/ml) with the color bar referring to the relative temperature.

Carbon nanomaterials have good light absorption properties in the visible wavelength range. Therefore, Cobalt at carbon NPs can achieve high light absorption and have the potential to perform photothermal therapy (PTT) on tumors. To assess the potential of Cobalt at carbon NPs to achieve PTT against tumors in living mice, we established U87-MG tumors on the back of Balb/C nude mice. After the dorsal tumor volume reached 80 mm^3^, eight tumor-bearing mice were randomly divided into two groups and received the same amount of (100 μl) PBS and Cobalt at carbon NPs (1 mg/ml) via tail vein. After 6 h of injection, the tumor area was irradiated with an 808-nm laser (1 W/cm^2^) for 5 min. At the same time, these mice were thermally imaged with an infrared thermal imager, and temperature changes were recorded (Figure 6). Under 808-nm laser irradiation, tumor temperature of mice injected with PBS only increased 9.2°C. In contrast, the tumor site temperature of mice injected with Cobalt at carbon NPs increased by 16.3°C after 808-nm laser irradiation for 5 min. At this time, the maximum temperature reached 48.8°C, which was sufficient to achieve photothermal ablation of the tumor (Lu et al., 2005; Day et al., 2012; MacDonald et al., 2014; Li et al., 2016; Shao et al., 2016). These results suggest that Cobalt at carbon NPs have the possibility of realizing PTT *in vivo* mouse tumors.

## Discussion

MRI, providing both anatomic and functional information, is one of the most powerful noninvasive imaging methods to image the whole human body in clinical diagnosis and prognosis. PAI is a new technique that depends on the contrast generated by the optical absorption property of the tissues. Compared with traditional optical imaging, PA imaging shows remarkably improved imaging depth, owing to the excellent soft tissue penetration ability of sound, and is capable to achieve high resolution imaging in deep-seated tissue successfully. Cobalt at carbon NPs with magnetization and optical absorption properties may thus be used as contrast agents for PAI and MRI contrasts simultaneously. When Cobalt at carbon NPs can orientation to the tumor site, preoperative and intraoperative imaging of tumor could be realized. Although several kinds of dual-modality PAI and MRI probes have been developed (Kircher et al., 2012; Bouchard et al., 2009), carbon shell–based nanoparticles (Cobalt at carbon NPs) with excellent biocompatibility are candidates for PAI and MRI dual-mode imaging. In addition, Cobalt at carbon NPs show good photothermal effect and have the potential of PTT for tumors. Herein, Cobalt at carbon NPs are concentrated at the tumor site through EPR effect. However, it has been reported that targeting molecules [such as RGD (Biscaglia et al., 2019; Liang et al., 2021; Cao et al., 2020) and folic acid (Prossnitz and Barton, 2014; De Marco et al., 2016)] that can specifically recognize tumor cells through surface connection can enhance the aggregation of nanoparticles at the tumor site and improve imaging and PTT ability. Hence, Cobalt at carbon NPs can probably be a candidate multifunctional nanodrug for dual-mode image-guided tumor PTT in the future. Detailed pharmacokinetic, *in vivo* toxicity, and PTT studies will be carried out in the next step.

In addition, to promote the clinical application of Cobalt at Carbon NPs, further exploration can be made from the following aspects: 1) To promote the clinical application of Cobalt at carbon NPs, first of all, it is necessary to ensure that their biocompatibility and biosafety are high enough, and further verification of the biocompatibility of new Cobalt at carbon NPs in organisms is required. 2) Add targeted molecules to improve the targeting ability, so that nanoparticles can specifically gather to the tumor site. 3) Studies have shown that the larger the size of the nanoprobe, the higher its uptake by the liver and the smaller the amount of the nanoprobe reaching the tumor site. High uptake of contrast agents by tumors can be achieved by reducing the size of nanoparticles.

## Conclusion

In summary, Cobalt at carbon NPs were successfully developed for the first time as a novel probe for MRI and PAI. The nanoprobes were obtained by coating carbon onto the cobalt nanoparticle. Cobalt at carbon NPs possess both ferromagnetic and optical absorption properties, which could be used for enhancing both MRI and photoacoustic signals both *in vitro* and *in vivo*. After intravenous injection of Cobalt at carbon NPs, Cobalt at carbon NPs can accumulate and retain in tumors, allowing for a noninvasive tumor imaging using by MRI and PAI system. With Cobalt at carbon NPs, it is possible to institute a multiscale complementary imaging protocol that MRI can screen to identify the location of tumor for preoperative planning, and high spatial resolution PAI provides subsequent precise blood vessel morphology and tumor imaging from the surface to the depths to accurately guide tumor resection.

## Methods and Materials

### Materials

Cobalt at carbon NPs were purchased from Sigma-Aldrich Co. Ltd. (Shanghai, China). Phospholipid–poly(ethylene glycol) (PL-PEG, MW of PEG = 5,000) were purchased from JenKem Technology Co. Ltd. Fluorescein isothiocyanate–annexin V, propyl iodide, and calcein acetoxymethyl ester were obtained from Kumamoto Dojindo Laboratories, Japan. All chemicals purchased are analytical grade and can be used directly without further purification. All the water used in the experiment is high-purity deionized water (resistance >18 MΩ cm). Animal handling procedures and animal care and Use are in full compliance with the relevant guidelines of the Animal Care and Use Council.

### PL-PEG–Functionalized Cobalt at Carbon NPs

Through the non-covalent functionalization of phospholipid-polyethylene glycol, a stable aqueous suspension of Cobalt at carbon nanotubes was prepared. The hydrocarbon chains of the phospholipids are adsorbed to the graphite shell through van der Waals and hydrophobic interactions, whereas the hydrophilic PEG chains extend to the water phase to achieve dissolution. Add PL-PEG-functionalized Cobalt at carbon nanotubes to the newly synthesized Cobalt at carbon nanotube solution (2 ml, 2 mg/ml), stir for 12 h to ensure that the polymer coating is complete. Then, centrifuging (8,000 rpm, 20 min), eliminate unbound PL-PEG molecules and prepare PL-PEG-functionalized cobalt at carbon nanotubes.

### Characterization Experiments

A HITACHI H-300 transmission electron microscope (TEM) was used to observe the samples with the parameters of 70-kV voltage and 70-pA current. Raman spectroscopy was used to detect the G and D bands of carbon. Use 1,064-nm laser excitation. After focusing at the center of the capillary, record the Raman spectrum of the sample. X-ray diffractometer (Bruker diffractometer, 3-kW Cu-Kα radiation) was used to analyze the Cobalt core/carbon shell nanocrystals.

### Magnetic Resonance Imaging *in vitro*

The Cobalt at carbon NPs were diluted with deionized water (18.2 MΩ resistivity) to prepare Cobalt at carbon NPs with different concentrations. The diluted core/carbon NPs sample is in an agar phantom. MRI uses 1.5-T imager (GE Signa HD, 1.5 T MR, GE Healthcare, Milwaukee, WI, United States) and 12.7 cm only accepts knee joint coils. The magnetic resonance coronal image is scanned with a fast spin echo T_2_ sequence [repetition time (ms)/echo time (ms) = 4,000/108, echo sequence length is 16], and an image with a matrix size of 256 × 256 is obtained.

### Photoacoustic Imaging System

A miniature laser (Laser-export Co. Ltd., Moscow, Russia), with a working wavelength of 532 nm, a pulse width of <10 ns, and a repetition frequency of up to 30 kHz, is used as the excitation light source for the PAI system. After the light passes through the scanning lens and the tube lens, it is focused by the objective lens and illuminates the sample under test. The average energy density of the laser was set to <20 mJ cm^−2^. After that, the PA signal generated by the tested sample is received by an ultrasonic transducer with a center frequency of 10 MHz and a bandwidth of −6 dB (China Doppler Electronic Technology Co., Ltd.). The photoacoustic signal is recorded by the computer through the data acquisition card. The data acquisition card has a sampling rate of 200 M samples/s. The two-dimensional scanning table is driven by a computer-controlled ultrasonic motor. The computer controls the ultrasonic motor to move the sample and analyzes the photoacoustic signal generated by each scanning point. Then, the PA image in the region of interest can be obtained.

### Photoacoustic Imaging *in vitro*

The synthesized nanoparticles were diluted with deionized water (18.2 MΩ resistivity) to prepare Cobalt at carbon NPs with different concentrations. The diluted core/carbon shell nanoparticle sample is embedded in an agar phantom. PAI is obtained through a PAI system.

### Cell Viability Assays

The cell cytotoxicity of Cobalt at carbon NPs was tested using the colorimetric tetrazolium base to detect CCK-8. U87-MG cells were seeded in a 96-well microtiter plate (5 × 10^3^ per well, 100 µl). The solutions of samples at 0 μg/ml (PBS), 5 μg/ml, 10 μg/ml, 50 μg/ml, 100 μg/ml 200 μg/ml, and 500 μg/ml were incubated for 24 h. After that, the cell viability was tested according to the CCK-8 assay. Each experiment was conducted in five independent parallel groups.

### *In vitro* Cell Uptake

For *in vitro* cell uptake test, U87 cells were seeded into a confocal cell culture dish (1 × 10^5^ cells/well) for 24 h, and then, the initial medium was replaced with a new serum-free medium, including Cobalt at carbon NPs–Cy5. Five and PBS, incubate for 3 h, wash with PBS three times, and observe the morphology with a confocal microscope (ZEIESS LSM SH120, Germany). Then, the samples (containing 10,000 cells) were run on a FACSCanto II flow cytometer (Becton Dickinson, Mountain View, CA, United States).

### Animal Culture

Male BALB/c nude mice were purchased from the Provincial Animal Center (Guangzhou, China), 3–4 weeks old, under the standard breeding conditions.

### Animal Models

The PBS dispersion of U87 MG cells (1 × 10^6^, 100 µl) was injected into the back area of Balb/c mice. After 1 week, the tumor grew to about 80 mm^3^ in size, thus successfully constructing a typical tumor transplantation model on the back of the tumor.

### MRI and PAI *in vivo*

Glioma-bearing mice (tumor volume was 80 mm^3^) were recorded images of the T2-weighted imaging and PAI (*λ* = 532 nm, pulse duration = 10 ns, and pulse energy = 10 mJ cm^−2^). Then, the mice were injected with 100 μl of Cobalt at carbon NPs solution (200 μg/ml). Six hours later, the animals were anesthetized with isoflurane, and MRI and PAI were performed.

## Data Availability

The raw data supporting the conclusions of this article will be made available by the authors, without undue reservation.
